# Factors Associated with Inappropriate Use of Antibiotics Among Animal Health Professionals in Selected Districts of Rwanda, 2021

**DOI:** 10.1007/s44197-024-00192-x

**Published:** 2024-02-26

**Authors:** Denyse Mugwaneza, Edson Rwagasore, Ziad El-Khatib, Pierre Dukuziyaturemye, Jared Omolo, Olivier Nsekuye, Samuel Rwunganira, Maximillian Manzi

**Affiliations:** 1 Rwanda Biomedical Center (RBC), Kigali, Rwanda; 2 Department of Global Public Health, Karolinska Institutet, Stockholm, Sweden; 3 University of Rwanda, Kigali, Rwanda; 4 Centers for Disease Control and Prevention (CDC), Kigali, Rwanda; 5 African Field Epidemiology Network (AFENET), Kigali, Rwanda; 6 Rwanda Agriculture and Animal Resources Development Board, Kigali, Rwanda

**Keywords:** Antibiotic resistance, Public health, Animal health professionals

## Abstract

**Background:**

Antibiotic resistance is a global health concern. Humans can acquire antibiotic resistance through human-to-human transmission, from the environment, via the food chain, and through the contact with animals. The National Action Plan on antimicrobial resistance 2020–2024 highlights the prudent use of antibiotics in veterinary activities as the key element in keeping antibiotics effective. We determined the factors associated with misuse of antibiotics among animal health professionals in Rwanda.

**Methods:**

This was a cross-sectional study that enrolled animal health field professionals from five districts, where stratified random sampling was used to select one district by each province of Rwanda. Structured questions were used during face-to-face interviews. The misuse of antibiotics was defined as the use of antibiotics for reasons other than treatment, the non-completion of required courses, or the use of a high dose (i.e., an overdose) of antibiotics. We collected socio-demographic data of respondents, as well as elementary knowledge and perceptions on veterinary antibiotics and antibiotic resistance. A backward stepwise logistic regression model was used to identify the factors that were predictive of the inappropriate use of antibiotics.

**Results:**

There were 256 respondents to the survey. Of those, 198 were male and 58 were female. Almost three quarters of respondents (*n* = 174/256; 68%) reported the misuse of antibiotics at least once in the previous 12 months. The final logistic regression analysis identified the following factors to be predictive of antibiotics misuse: aged ≤ 24 years (aOR 0.92; 95% CI [0.88, 0.96]; *p* < 0.001); low trust in veterinary antibiotics available in the local market (aOR 8.45; 95% CI [4.18, 17.07]; *p* < 0.01), insufficient knowledge about basic understanding of antibiotics and antibiotic resistance (aOR 2.78; 95% CI [1.38, 5.58], *p* < 0.01) and not acquiring any continuing education (aOR 1.97; 95% CI [1.02, 4.19]; *p* = 0.04).

**Conclusions:**

This study identified inadequate perceptions of proper antibiotic use among animal health professionals. There is a need for continuous education on appropriate antibiotic use among animal health professionals to lessen the negative impact of antibiotic resistance on public health security.

## Introduction

Globally, 700,000 deaths are attributed to antibiotic resistance each year, and this number could increase to 10 million by 2050 [1, 2]. The misuse of antibiotics includes the use (administration, dispensing or prescription) of antibiotics for reasons other than treatment, not completing prescribed courses and/or incorrect doses of antibiotics, either too high (overdose) or too low (underdose) doses of antibiotics.

The misuse of antibiotics in humans and animals has been identified as the leading cause in the development of antibiotic resistant pathogens [3–5]. Interaction between animals, humans and the environment, stimulates resistant gene shifts across several species and make antibiotic resistance a major global health concern [6–10]. The One Health approach tends to be a comprehensive and effective strategy in the global fight against antimicrobial resistance (AMR). This approach acknowledges the link between environment, animal health and public health; and highlight the need for a strategy that interlink these sectors [11].

Antibiotic resistance is known as the mechanism by which bacteria develop the ability to resist the action of an antibiotic that previously affected them [12]. Resistant bacteria can emerge from food-producing animals and be transferred to humans either through the environment or the food supply, or through direct contact with animals [13]. In 2011, US livestock producers purchased 29.9 million pounds of antimicrobials compared to the 37 million pounds used for nontherapeutic agricultural purposes in the European Union [14]. In the EU, 20% of human infection is caused by antibiotic-resistant bacteria [15]. Studies conducted in Malaysia and Bangladesh found that the misuse of antibiotics was associated with the participants' knowledge of antibiotics and their demographics [16, 17].

In sub-Saharan African states, antibiotics may be traded in the absence of any medical approval [18]. On the animal side, the lack of strict control measures could lead to malpractices, such as farmers purchasing and administering antibiotics without any veterinary prescription or supervision, or there is non-completion of recommended antibiotics course, administration of high doses, or frequent use of broad-spectrum of antibiotics, among other inappropriate uses of antibiotics [19]. To solve the rising urgency of antibiotic resistance in both humans and animals in sub-Saharan African states, multisectorial collaborations are needed. Unfortunately, these are still missing due to the lack of coordination between veterinary and public health surveillance systems [20].

In Kenya, over 200 genes conferring antibiotic resistance have been identified, primarily associated with respiratory and enteric infections in livestock. These genes can be disseminated between animals and are transmissible to humans [21]. Antibiotic resistance is an increasing public health challenge in Rwanda Inappropriate use of antibiotics has been documented in animal farms in Rwanda [22]. Adequate and effective use of antibiotics can help in preventing the occurrence of new life-threatening diseases causes by multi-drug resistant bacteria [23, 24].

Animal health professionals are indispensable in containing antibiotic resistance and safeguarding the efficacy of antibiotics for both human and veterinary medicine [25, 26]. Knowledge about disease prevention and prudent use of antibiotics among animal health professionals could be an effective approach for containing antimicrobial resistance [27]. However, little is known about the level of knowledge and practices of animal health professionals regarding prudent use of antibiotics in Rwanda. Therefore, the main objective of this study was to find the factors contributing to inappropriate use of antibiotics among animal health professionals.

## Methodology

### Study Setting and Design

This was a cross-sectional study that was conducted among field animal health professionals in Rwanda from January to March 2021. Rwanda, a small landlocked country located in East Africa, has a population of about 12.5 million people. It is bordered by the Democratic Republic of Congo on the west, Tanzania on the east, Uganda on the north, and Burundi on the south [25]. Rwanda’s economy and the population’s livelihoods are reliant on natural resources such as water, land, plants and animals [26]. The agriculture and livestock sectors have been prioritized for national sustainable development [28], and the size of the livestock sector is significantly increasing [29]. Agropastoral practices accompanied by excessive chemical use have been identified as activities leading to pollution [30, 31]. The country is hilly and mountainous with an altitude that varies between 900 and 4500 m [32]. Infectious, or communicable, diseases are the leading cause of sickness and death in Rwanda, accounting for 90% of all reported medical consultations in health facilities [33]. Intersectorial communication between environmental, public health and animal professionals has committed to work together in Rwanda [34].

This study used data from five districts in Rwanda including Nyarugenge of Kigali, Bugesera in the Eastern Province, Muhanga in the Southern Province, Gicumbi in the Northern Province, and Rubavu district in the Western Province. The stratified random sampling was used to select districts of study.

Nyarugenge, a district in Kigali, is where the city center is located. The district is bordered by Nyabarongo River which runs along much of the district’s western and southern boundaries. The economic inactivity is calculated at 22% and agriculture (cultivation and livestock) activities only occupy 17% of the population. The district covers an area of 134 km^2^ and inhabited by 284,561 people [35]. Bugesera is a District of the Eastern Province. The region is characterized by low precipitation. The population density is calculated at 282/km^2^. Muhanga is one of eight districts of the Southern Provinces lying on 647.7 km^2^ of land. The population density is estimated at 568.63 people/km^2^ [36, 37]. Rubavu one of the seven districts in the Western Province covering 388.3 km^2^ of land with a population density of 1,038 people/km^2^ while Gicumbi is one of five districts of the Northern Province with a surface of 829 km^2^ and a population density of 549 people/km^2^ [38].

## Study Population

This study included all animal health professionals who were working in the selected districts.

### Inclusion Criteria

The study targeted all animal health professionals working in the field in the Nyarugenge, Bugesera, Rubavu, Muhanga and Gicumbi districts.

### Exclusion Criteria

Any animal health professional living in selected districts but not working veterinary activities or working veterinary practice only outside of the study area was not eligible to participate in this study.

### Data Collection

A structured questionnaire, prepared in English and translated into Kinyarwanda, was administered during data collection. Prior to the study, 30 participants who were registered members of the Rwanda Council of Veterinary Doctors working outside of the research area participated in a pilot study in order to verify the adaptability and reliability of the questionnaire. A fundamental aspect of producing valid findings is the ability to generalize from the study population to the broader population in research [39]. Participants were visited at their working places. The tool was paper-based and data were entered into an Excel spreadsheet promptly after collection in the field.

### Sample Size and Sampling Strategy

This study used a randomized, consecutive sampling strategy. The consecutive sampling procedure involved randomly selecting one district in each province. From the selected district, all field animal health professionals were invited to participate in the research. The list of professionals working in the districts was received from district animal resources officers.

### Study Variables

#### Dependent Variable

The dependent variable was the use of antibiotics and was considered to be a dichotomous variable: appropriate use and inappropriate use.

Inappropriate use of antibiotics consisted of the use (administration, dispensing or prescription) of antibiotics for reasons other than treatment, non-completion of required doses, and/or use of overdoses doses of antibiotics by animal health professionals in the last 12 months from the date of interview. Appropriate use was defined as the appropriate use of antibiotics in the conditions of specific treatment, adequate completion of required doses and not exceeding the recommended dose of antibiotics.

#### Independent Variables

- Age of respondents, collected under four categories: (1) ≤ 24 years (reflecting the young professionals), (2) 25–35 years (reflecting the intermediate professionals), 36–50 years (reflecting the advanced professionals) and ≥ 50 years (reflecting the senior and the professionals near retirement).
- Gender was categorized into male and female.
- Working district referred to the location, where the participant worked.
- Educational level referred to the years of formal instruction acquired and completed successfully. It was grouped in three categories: A0 for the people who completed their bachelor’s degree either in Veterinary Medicine or Animal production, A1 for the people who completed their 3 year post-secondary studies in animal health, animal production or veterinary technology, and A2 for the people who completed secondary learning in animal health.
- Knowledge was measured using a set of four questions aimed to investigate if the respondents knew: (a) the best definition of antimicrobial resistance, (b) whether they knew that antibiotics kill both pathogenic bacteria and commensal bacteria, (c) the meaning of the term “one health”, and (d) the spread of antibiotic-resistant bacteria to other animals or humans. The scoring procedure was to give 1 mark for a correct answer and 0 marks for an incorrect response. The respondents who failed to correctly respond to at least three of four questions were classified as having insufficient knowledge.
- The level of trust in antibiotics on the market was measured by asking the respondents to base their responses on field observations regarding the quality of veterinary antibiotics available in their working district”. Responses were categorized as “very low”, “low” and “neutral” were classified into “low level”, while “high” and “very high” were classified into “high level”.
- Continuing education was defined as training acquired by a field animal health professional other than that of formal teaching system in the last 3 years, whereby the use of antibiotic or antibiotic resistance was a major focus. This included conferences, workshops, one-on-one consultations, self-directed learning, and web-based learning among others. For those who were attending formal school after March, 2019, the knowledge received during that period in formal learning was considered as continuing education.
- Employer was categorized as: “public service” for animal health professionals who are engaged and paid monthly by the government and “private practices” for those who were working for farmers as their own business.

#### Definitions of Key Terms

- Animal health professional was defined as someone qualified for implementing activities that are directly or indirectly linked to animals, their products and by-products, which assist in safeguarding, maintaining, and improving animal health with requiring at least A2 level of education [40].
- Antibiotic administration is the act of introducing an antibiotic to an animal through any route of administration, including intravenous, intramuscular, or oral, among others.
- Antibiotic dispensing was defined as the action of selling the medicine to the animal owner for home administration, which should be in accordance with a medical prescription.
- Prescription consisted of an instruction written by an animal health professional authorizing the animal owner to receive an antibiotic [41].
- Appropriate antibiotic treatment is defined as the use of antibiotics specifically for treating animals with diseases caused by bacterial infections.

### Data Analysis

Univariate analysis was plotted with frequencies and percentages in tables and charts; bivariate analysis was computed with OR and CI (95%) between each covariate and the outcome variable. Later, all significant variables within the bivariate model were put into the full backward logistic regression model to compute the adjusted OR for the variables with statistically significant variables were considered as factors that predicted inappropriate use of antibiotics. All variables with *p* values ≤ 0.15 in the basic model were added into the backward selection multiple logistic regression analysis (the inclusion criterion was *p* < 0.05). This model was referred to as the final model. The following variables were included in the model regardless of *p* value: Gender; District; Type of employer; Level of trust of antibiotics available on market; Level of knowledge; and Continuing education. Data analysis was conducted using Stata 15 [42].

## Results

### Socio-demographic Characteristics

This study included 256 (84.2%) of 304 animal health professionals working in the field in Bugesera, Nyarugenge, Muhanga, Rubavu and Gicumbi districts who responded to the questionnaire. The remaining 48 were either not interested in participating in the research or were not available on the appointment date to respond to the questionnaire.

Among 256 respondents, 174 (67.96%) used antibiotics inappropriately, 198 (77.3%) were male and 58 (22.7%) were female, the ages ranged from 21 to 56 years (median of 32 years) with the mean of 34 years and the standard deviation of 8 years. Just over two fifths (41.0%) had been practicing animal health profession for 6–15 years. Most of the respondents (72.8%, 186) were working in private sector. Regarding education level, almost three quarters had completed secondary (A2) as the highest level of study in the field of animal health (Table 1).

**Table 1:** Characteristics of respondents

Characteristics	*N*	%	95% CI
Gender			
Male	198	77.34	[71.78–82.08]
Female	58	22.66	[17.91–28.21]
Age			
24 years and below	30	11.72	[8.30–16.29]
25–35 years	123	48.05	[41.95–54.19]
36–50 years	96	37.50	[31.75–43.62]
> 50	7	2.73	[1.30–5.64]
Level of education			
A2	181	70.70	[64.80–75.97]
A1	22	8.59	[5.71–12.72]
A0	53	20.70	[16.15–26.13]
Employer			
Public servant	70	27.17	[22.02–32.99]
Private practice	186	72.83	[67.00–77.97]
Working district			
Bugesera	50	19.53	[15.10–24.87]
Nyarugenge	42	16.41	[12.33–21.48]
Muhanga	55	21.48	[16.85–26.96]
Rubavu	50	19.53	[15.10–24.87]
Gicumbi	59	23.05	[18.27–28.63]
Level of knowledge			
Sufficient	82	32.03	[26.57–38.02]
Insufficient	174	67.97	[61.97–73.42]
Level of trust			
High	122	47.66	[41.57–53.80]
Low	134	52.34	[46.19–58.42]
Acquired continuing education			
Yes	105	41.02	[35.12–47.17]
No	151	58.98	[52.82–64.87]

Regarding the scientific statements, 35 participants (13.67%) were not aware that antibiotic use in one patient may weaken its effectiveness in the same individual in the future, the majority of the respondents (88.67%) were aware that antibiotic use in one animal may weaken its effectiveness for other animals, and 212 respondents (82.81%) were aware that a single course of antibiotics can cause antibiotic resistance. Pertaining to the capacity of health systems to maintain the status of population health in the face of antimicrobial resistance, 91.80% of animal health professionals believed that new antibiotics would be developed that address the problem of antibiotic resistance.

In terms of malpractices executed by animal health professionals, 116 respondents (45.31%) reported that they have prescribed an overdose of antibiotics in the past year. For the question related to non-completion of required antibiotics course, “yes” was indicated by 39 respondents (15.23%) respondents, and 91 (35.54%) respondents indicated they had provided antibiotics for reasons other than treatment.

Regarding the reasons that led to overdosing during antibiotics use, 116 respondents were asked to highlight the reasons influencing their decision to prescribe an overdose. Among these participants, 78 (67.24%) indicated that “they thought the quality of antibiotics was low, so the increased dose would help to improve the curative ability of antibiotics”; 21 (18.10%) indicated “with experience, they noted the recommended dose written on the leaflet did not work”, while 8 (6.89%) indicated they overdose antibiotics based to information received from their colleagues.

Looking at prescribing antibiotics for reasons other than treatment, among 91 who confirmed the malpractice, the intended purpose was for “disease prevention” in 65 (71.42%), “growth purpose” in 9 (9.89%), “small number of animals to be sick then treat all” in 15 (16.49%) while 2 respondents (2.20%) reported “other reasons” (Table 2).

**Table 2:** Bivariate analysis, antibiotic use and characteristics of respondents

Reason attributed to the malpractice	Total	*N*	%
*Malpractice I: Overdose*			
Increase of dose will help to adequate cure		**78**	**67.24**
I noted that the recommended dose to the leaflet do not work		**21**	**18.10**
I referred to the information from my colleagues	**116**	**8**	**6.89**
*Malpractice II: Prescribe antibiotics for other reason than treatment*			
Disease prevention		**65**	**71.42**
Growing purpose	**91**	**9**	**9.89**
Small number of sick animals, then you treat the whole farm		**15**	**16.49**
Other		**2**	**2.20**
*Malpractice III: non completion of required antibiotics shots*			
Animal was recovered		**23**	**58.97**
Purchasing power of farmer to buy necessary quantity of antibiotics	**39**	**6**	**15.38**
Farmer refused to pay the service for next shot		**5**	**12.82**
Lack of time		**3**	**7.69**
Fear of transport cost		**2**	**5.12**

Bold values indicate *p* < 0.05

### Factors Associated with Inappropriate Use of Antibiotics Among Animal Health Professionals

Multivariate analysis showed that knowledge level regarding antibiotics and antibiotic resistance was significantly associated with inappropriate use of antibiotics, whereby the animal health professionals with insufficient knowledge were found to be three times more likely to use antibiotics in an inappropriate way (OR 2.78; 95% CI [1.38, 5.58]; *p* < 0.01). Regarding the level of trust in the quality of veterinary antibiotics available to the market, animal health professionals with low trust were eight times more likely to use antibiotics inappropriately in animals than those who expressed high trust (OR 8.45; 95% CI [4.18, 17.07]; *p* < 0.01). Not acquiring any continuous education was two times more likely to lead to antibiotic misuse (OR 1.97; 95% CI [1.02, 4.19]; *p* = 0.04). The other variables were not statistically significant (Table 3).

**Table 3:** Factors associated to antibiotic use among veterinarians

Characteristics	Basic model		Final model	
	OR (95%)	*p* value	aOR (95%)	*p* value
Gender^a^				
Men	1 (Ref)		1 (Ref)	
Women	0.55 (0.24–1.22)	0.14	0.55 (0.25–1.23)	0.14
Education				
A0	1 (Ref)			
A1	0.89 (0.25–3.15	0.85		
A2	1.20 (0.54–2.67)	0.66		
Age	0.92 (0.88–0.96)	< 0.01	0.92 (0.88–0.96)	< 0.001
District^a^	0.91 (0.72–1.15)	0.42	0.91 (0.72–1.15)	0.42
Employer				
Public servants	1(Ref)			
Private practice	1.77 (0.84–3.73)	0.13	1.88 (0.92–3.84)	0.09
Level of trust of antibiotics on market^a^				
High	1 (Ref)			
Low	**8.59 (4.23–17.43)**	** < 0.01**	**8.45 (4.18–17.07)**	** < 0.01**
Level of knowledge^a^				
Sufficient	1 (Ref)			
Insufficient	**2.75 (1.36–5.55)**	** < 0.01**	**2.78 (1.38–5.58)**	** < 0.01**
Continuing education^a^				
Yes	1			
No	**1.97 (1.02–3.80**	**0.04**	**1.97 (1.02–4.19)**	**0.04**

Bold values indicate *p* < 0.05

^a^Variables forced into the final model

## Discussion

This cross-sectional study was conducted in one district in each province in Rwanda. There were 256 animal health professionals who responded to the questionnaire, resulting in a response rate of 84% from professionals working in Bugesera, Gicumbi, Muhanga, Nyarugenge and Rubavu. Of these respondents, 174 (67.97%) had insufficient knowledge regarding antibiotics and antibiotic resistance This is consistent with studies conducted among veterinarians and para-veterinarians, where 61.25% of respondents in Bhutan and 52.6% in Nigeria have shown to have poor knowledge related to antibiotics and antibiotic resistance [43, 44]. This could be due to deficient awareness on antibiotic resistance among animal health professionals. Only 105 (41.02%) of respondents had attended at least one antibiotic resistance related continuing education in the previous 3 years. Animal health professionals with insufficient knowledge were more likely to misuse antibiotics than their counterparts with sufficient knowledge. This finding is consistent with other studies conducted in Nepal [45] and in Korea confirming the influence of knowledge on antibiotic resistance with inappropriate use of antibiotics [46]. This finding highlighted the importance of improving awareness regarding the adequate use of antibiotics among animal health professionals to help contain the burden of antibiotic resistance.

Inappropriate use was mentioned by 174 (67.96%) respondents. This is high compared to other studies, where only half of participants were inappropriately prescribing antibiotics [47, 48].

There is a need to develop detailed documents such as AMR surveillance strategy and an antimicrobial stewardship strategic plan to support the implementation of the national action plan on antimicrobial resistance 2020–2024. Pertaining to non-completion of recommended doses of antibiotics, among the 39 respondents that answered with a “yes”, 23 (58.97%) reported they didn’t complete all the doses because the animals receiving the antimicrobial treatment did recover prior the end of the treatment regimen; 6 (15.38%) indicated that the farmers were incapable to buy necessary quantity of antibiotics for completing required doses; 5 (12.82%) indicated that the animals’ owners refused to pay for the service of the remaining doses; 3 (7.69%) indicated lack of time as the reason; and 2 (5.12%) did not complete recommended doses due to the concerns about the cost of transport. This contrasts with another study conducted in South Africa, which identified the cost of antibiotics as the primary factor influencing decisions related to antibiotic use [49], similar to findings from a study in Australia [50].

Being a junior professional was identified as the factor contributing to the prudent use of antibiotics, which is in contrast with the study conducted in Ethiopia, where young people misused the antibiotics more often [51]. Participants with low trust in the quality of veterinary antibiotics available to the market were more likely to inappropriately use antibiotics. This is not surprising, because the more one trusts the quality of a drug, the more they follow the written instructions related to the drug; once one has low trust in the quality, they tend to provide a higher quantity (overdose) in order to improve the efficacy of the drug. This is similar to a study conducted in Brazil [52].

The study did not establish a statistically significant influence of gender, education level or type of employer as far as the inappropriate use of antibiotics was concerned. This is in contrast with the study conducted in New Zealand and others conducted in developing countries [53, 54].

The data used in this research was obtained directly from respondents. Data collectors were trained on the data collection tool prior to the study, and a trial of the tool was conducted in the field to ensure the feasibility of the interview. Nevertheless, this study had limitations. We were not able to observe the antibiotic use behavior of animal health professionals directly when they were working in the field, nor could we assess whether the recommended doses were completed or if antibiotics were used exclusively for treatment and in adequate doses. Despite the limitations related to self-reported data, the study's design incorporated measures to enhance data reliability. The results thus offer a valuable perspective on the studied population, though they should be considered with an understanding of the potential for response bias.

## Conclusion and Recommendations

Inappropriate use of antibiotics by animal health professionals in Rwanda was relatively high. Knowledge about antibiotics was a predictor of their misuse by veterinary professionals. The animal health professionals were prone to administering overdoses thinking that the curative power of antibiotics should be improved by elevating the quantity of the administered drug. The level of trust in the quality of antibiotics available on the veterinary drug market was significantly predictive of inappropriate use of antibiotics.

The professionals who did not participate in any continuing education programs were most likely to misuse the use of antibiotics in animals. This could be because they were not aware of the interaction of animal health and public health as the new concepts such as one health approach came in along the time.

Therefore:

- The Ministry of Agriculture should enhance animal health professionals’ knowledge the effective and prudent use of antibiotics.
- The Ministry of Health should collaborate with the Ministry of Agriculture to establish clear guidelines on antibiotic use and the implementation of antimicrobial stewardship.
- Local leaders should enforce the implementation of available rules and regulations related to the prevention of antibiotic resistance.
- There is a need for continuous education among animal health professionals and expanded focus to reflect on the impact of their practice regarding one health and public health security.
- Further research is needed in order to determine the level of antibiotic resistance in animals and its impact on public health.

## Data Availability

All relevant data are included in this manuscript. However, used dataset may be shared upon reasonable request to the corresponding author.

## Abbreviations

%: Percentage
A0: Bachelor’s degree
A1: Advanced degree
A2: Humanity degree/secondary education
CI: Confidence interval
N: Nombre
OR: Odds ratio
US: United State
EU: European Union
